# Bedside Percutaneous Tracheostomy versus Open Surgical Tracheostomy in Non-ICU Patients

**DOI:** 10.1155/2014/156814

**Published:** 2014-01-12

**Authors:** Evgeni Brotfain, Leonid Koyfman, Amit Frenkel, Michael Semyonov, Jochanan G. Peiser, Hagit Hayun-Maman, Matthew Boyko, Shaun E. Gruenbaum, Alexander Zlotnik, Moti Klein

**Affiliations:** ^1^Department of Anesthesiology and Critical Care, Soroka Medical Center, Sderot Rager, Beer Sheva 84100, Israel; ^2^Department of Medical Management, Soroka Medical Center, Ben-Gurion University of the Negev, Beer Sheva, Israel; ^3^Department of Economic Management, Economist, Soroka Medical Center, Ben-Gurion University of the Negev, Beer Sheva, Israel; ^4^Department of Anesthesiology, Yale University School of Medicine, New Haven, CT, USA

## Abstract

Percutaneous bedside tracheostomy (PBT) is a one of the common and safe procedures in intensive care units through the world. In the present paper we published our clinical experience with a performance of PBTs in the regular ward by intensive care physicians' team. We found it safe and similar outcome in comparison to open surgical tracheostomy method in operation room by ENT team. The performance of PBT in the regular ward showed potential economic advantages in saving medical staff and operating room resources.

## 1. Introduction

Over the last two decades, percutaneous bedside tracheostomy (PBT) has been frequently performed in critically ill patients [1, 2]. Compared with the open surgical technique, PBT has been implemented for similar clinical indications such as protection of the larynx and the upper airway, as well as weaning from prolonged mechanical ventilation [3, 4].

PBT was demonstrated to be as safe as the conventional surgical approach in most critically ill patients [5, 6]. Moreover, the overall rate of surgical bleeding and stomal infection was lower in the bedside technique compared with the open approach. Both techniques have been shown to have similar mortality rates in the Intensive Care Unit (ICU) and in the inpatient wards [7]. However, the ventilation times and length of stay in the ICU following PBT were demonstrated to be significantly shorter [8].

Bedside tracheostomy may be especially beneficial for patients who require prolonged mechanical ventilation. Performing a bedside tracheostomy has become common practice in ICUs in Israel. PBT can be performed quickly and safely by an ICU team trained and familiar with the procedure (anesthesiologists, intensive care physicians, etc.) [6] and does not require the use of the operating room facilities.

Not surprisingly, most bedside tracheostomies in the ICU are performed by intensive care physicians, whereas only a minority was performed by ear, nose, and throat (ENT) surgeons. In 2007, we published data reflecting our clinical experience of PBT procedure performed by intensive care physicians in the ICU [9].

In Israel, most mechanically ventilated adult patients are admitted to inpatient wards other than the ICU due to a shortage of ICU beds. To circumvent this problem, an Outreach PBT program was initiated by critical care physicians at our institution. We anticipated that there would be economic advantages regarding the bedside procedure performance.

## 2. Materials and Methods

In this study, we retrospectively examined clinical data over six years and compared clinical data and economic analysis associated with the Outreach ICU PBT procedures performed in non-ICU patients at our tertiary care center and the open surgical tracheostomy technique performed by ENT surgeons in the operating theater.

### 2.1. Study Design

This is an observational, retrospective study performed in university teaching hospital.

### 2.2. Study Comparators and Population

The Human Research and Ethics Committee at Soroka Medical Center in Beer Sheva, Israel, approved this study. We collected clinical data from all cases of tracheostomy performed at Soroka Medical Center between January 2006 and June 2012. Soroka Medical Center is a tertiary care facility with 1100 inpatient beds, including 20 (1.8%) ICU beds. Data from the percutaneous Outreach ICU program and open surgical tracheostomies performed in the operative theater were extracted from the Operating Room Registry.

#### 2.2.1. Exclusion Criteria

Open surgical tracheostomies performed on pediatric patients as well as elective tracheostomies planned and performed by ENT surgeons were excluded.

#### 2.2.2. The ICU Outreach Team Protocol for Percutaneous Dilatational Tracheostomy

All Outreach ICU procedures were performed according to our Outreach Team Protocol. In the first step, the treating team of the regular ward made the decision about tracheostomy. Prior to performing the procedure, patients were presented with written consent that included the clear indication for tracheostomy (airway protection and weaning from prolonged mechanical ventilation). Then, all patients were examined by an intensive care physician prior to the procedure and clinical contraindications were excluded (anatomical neck limitations, significant coagulopathy, morbid obesity, presence of a pulsatile artery over the surgical area, and inability to identify the cricoid cartilage).

Our ICU Outreach Team consists of 3 physicians: one staff intensive care physician with at least two-year experience in performing the procedure, an assistant (resident or ICU fellow) physician, and an anesthesiologist. A registered nurse from the ward also assists in the procedure. The Outreach Team uses standardized equipment for percutaneous dilatational tracheostomy (Table 1), which is prepared and checked prior to beginning the procedure.

All PBTs are performed on intubated patients, under adequate sedation and muscle relaxation and with administration of 100% oxygen. Patients are monitored with an electrocardiogram, noninvasive oscillatory blood pressure measurement, pulse oximetry, and capnography. Equipment for emergent reintubation, large size suction, and a mechanical ventilator are prepared and checked before the start of the procedure. The staff ICU physician is responsible for rechecking all equipment prior to beginning the procedure. Prior to performing the procedure, the availability of an ENT surgeon is confirmed in the event of complications. A chest X-ray is routinely done after PBT. After performance of tracheostomy the critical care team does not continue to follow the patient on the ward.

#### 2.2.3. Location

The ICU Outreach Team was approved to perform PBT in all medical wards (internal medicine, neurology, neurosurgery, cardiothoracic, and intensive cardiological care unit (ICCU)) of Soroka Medical Center with the exception of the neonatal and pediatric departments.

#### 2.2.4. Methods

All percutaneous Outreach ICU tracheostomies were done using the Portex Griggs method [10] without bronchoscopic assistance. An ENT team in the operating room using the classic approach performed all open surgical tracheostomies.

### 2.3. Data Collection

#### 2.3.1. Variables and Measures

The demographic data, reasons for hospital admission, indications for tracheostomy, length of resources utilization, complication rate, and success rate of weaning from mechanical ventilation, in-hospital mortality, and economic rationality of both methods were collected and analyzed from patients' records in both groups.

#### 2.3.2. Economic Analysis

The cost-effective analysis of both the Outreach ICU and intraoperative procedures included the staff and operating room resources, tracheostomy set cost, and fee charges. Length of resources utilization was defined as the time in minutes to perform PBT by the Outreach ICU Team including the time of the patients and set-up position (group 1) and the time from transferring the patient to the operating room until the patient returned to the ward (group 2). It should be noted that the performance of tracheostomy in the operating room was always associated with additional events (including transferring patients from the ward to the operating room, operating room cleaning and preparation, and anesthetic management) that lead to unavoidable lost time and subsequent inability to utilize the operating theater for other procedures. Furthermore, from the moment the hospital patient transport service worker is sent to bring the patient to the operating theater, the room is prepared for the ventilated patient and no other patient may undergo a surgical procedure in that room (Table 3). This time was included in the total time taken for an open surgical tracheostomy in the operating room.

Staff and operating room resources included “operating room time” (in OR) cost, dressing and sterile set materials in operating room and in the ward, and staff physician time cost per hour (ENT and anesthesia team in OR and Outreach ICU Team on the ward).

All economic analysis was estimated by the Hospital Financial Expert Service Group.

All costs were presented as a mean ± SD.

### 2.4. Statistical Analysis

Statistical evaluation of the results was done with the SPSS 18 package (SPSS Inc., Chicago, IL, USA). Normally distributed data and continuous variable are presented by mean ± standard deviation (SD). Statistical comparisons between the two study groups for parametric data were conducted using Student's *t*-test. Nonparametric data was analyzed with a 2 × 2 contingency table and a Fisher's exact test. Statistical significance was defined as *P* < 0.05.

## 3. Results

A total of 685 tracheostomies were performed at our institution over a six-year period and included elective, semielective, and emergent cases. 70 patients (group 1) underwent percutaneous bedside Outreach ICU tracheostomy by the intensive care physicians' team and 615 patients underwent open tracheostomy by ENT surgeons in the operating room. After applying the exclusion criteria, 443 patients were included in the open tracheostomy group (group 2).

There was no difference in age distribution between the two study groups (Table 2). Patients in group 2 had a higher incidence of sepsis, whereas patients in group 1 had a higher incidence of acute stroke and intracranial hemorrhage (*P* < 0.05, Table 2).

The length of resources utilization was significantly shorter in the Outreach ICU group compared with the open surgical tracheostomy group (20 ± 8.5 min versus 77.5 ± 14.7 min, *P* < 0.0001, Table 3). There were no statistically significant differences in the rate of successful weaning, intraoperative complications, or mortality between groups (Table 3).

Two cases of accidental false-passage cannulation were encountered and successfully recannulated by the Outreach ICU Team during performance of the bedside procedure. Complications during the open surgical approach included significant intraoperative bleeding, accidental pneumothorax, and misplacement of the cannula (see Table 3 for details). There were no intraoperative complications that resulted in patient deaths. Intraoperative management of bleeding and pneumothorax included control of the bleeding and thoracic drainage, respectively.

### 3.1. Economic Rationale

We found significant economic advantages to performing PBT by the Outreach ICU Team compared with open surgical tracheostomy in operating room (Table 4). PBT was associated with reduced costs of medical staff resources compared to the open procedure (*P* < 0.0001). By examining the length of resources utilization between the two groups (Tables 3 and 4 for details), the bedside procedure may save significantly more operating room time annually. Thus, the annual economic analysis showed potential savings of approximately 50,000 US $ by better utilizing the operating room resources (total balance per procedure, 338 ± 10 US $ versus 561 ± 10 US $, *P* < 0.01Table 4).

## 4. Discussion

PBT has become a widely performed bedside procedure in patients in the ICU. Since its introduction in 1969 by Toy and Weinstein [11], multiple multicenter analyses and systematic reviews have been published comparing bedside tracheostomy to the standard surgical technique (described by Jackson in 1909) [7, 8, 10, 12].

In many countries, there are a limited number of ICU beds (1.8% of all beds in our hospital) [13]. Many patients who required mechanical ventilation are usually treated in the regular wards [14]. Moreover, there is a considerable delay in the performance of open tracheostomy in the operating room by ENT surgeons due to a lack of available operating theaters and the relative high demand for elective and emergency procedures. These delays may range in duration from two to 14 days. As such, this may result in a significant waiting time for tracheostomies performed by ENT surgeons, which may subsequently prolong the hospital length of stay, delay the weaning process [15–18], and increase patient morbidity [19]. Despite detailed and large number of studies about using percutaneous bedside tracheostomy (PBT) method in critical care units, we could not find any report about performance of PBT outside ICU in regular ward.

We found no differences in the intraoperative complication rate, weaning success, and survival rates between our Outreach ICU Team performance and the open surgical method in the operating room. In contrast to Outreach ICU PBT performance (group 1) where false passage cannulation occurred, intraoperative complications in open surgical tracheostomy group 2 were related to intraoperative bleeding, pneumothorax, and accidental high tracheostomy placement. However, none were associated with fatal feature.

Otherwise, the Outreach ICU Team required significantly less resources utilization time and was considerably cheaper than the open procedure in the operating room. Similarly, the Outreach ICU Team did not require the availability of an operating room or ENT team.

The cost-effective analysis of both procedures demonstrated significant savings with regard to operating room and staff resources in the PBT compared to open surgical approach. In contrast, the tracheostomy sets were more expensive in the Outreach ICU group, which has also been well described in the literature [20].

Our study has a number of limitations. We showed the prevalence of septic patients in open surgical tracheostomy group in contrast to acute neurological disturbances (acute CVA, intracranial bleeding) in Outreach ICU group. It might be explained by possible selection bias of use of retrospective data in the present paper. This might be controlled for in a randomized prospective study. Another limitation of our study is a major difference with respect to numbers in each group.

Also a precise economic analysis may be difficult to accurately analyze due to differences in time and logistics between procedures.

Future investigations might also include the detailed analysis of the operating room utilization resources benefit from saving charges and using free operating room time space for additional elective procedures, examination of delayed complications, length of ventilation, and length of hospital stay.

In present study, PBT has been demonstrated to be not only a safe procedure but also considerably cheaper than the open surgical method. We believe that the economical and clinical advantages of PBT method are worth considering in patients who require tracheostomy.

## 5. Conclusion

Performance of PBT in the wards should be considered safe if performed by physicians with the appropriate procedural skills. PBT may also prevent subsequent complications associated with prolonged tracheal intubation. We suggest that PBT may be more cost-effective in terms of reducing the length of procedure and need for surgical staff and equipment.

## Figures and Tables

**Table 1:** Standardized set for percutaneous tracheostomy. ICU Outreach Team, Soroka Medical Center.

Subject	Number of equipment
Surgical gown	3
Sterile gloves	3 pairs
Sterile towels	8–12
Set for percutaneous tracheostomy*	2
Skin and soft tissue dilator	1
Anesthesia medications**	1 set
Scissors	1
Tracheostomy report	1

*Usually, there are two different sizes of tracheostomy tubes in the set: size 9 for men and 8 for women.

**Anesthesia medications always include hypnotic agents, analgesics, and neuromuscular relaxants.

**Table 2:** Demographic data (mean ± SD, %).

	Group 1 (Outreach ICU) (*n* = 70)	Group 2 (open) (*n* = 443)	*P* value
Age (mean ± SD)	60.58 ± 22.5	62.4 ± 19.3	>0.05
Gender (male : female)	51 : 19	256 : 187	>0.05
Diagnosis on admission (%)			
Severe sepsis	2.9 (*n* = 2)	15.5 (*n* = 69)	<0.05**
Trauma	20 (*n* = 14)	18.7 (*n* = 83)	0.8
COPD exacerbation	8.5 (*n* = 6)	7.2 (*n* = 32)	0.8
Acute ischemic stroke	24.2 (*n* = 17)	7.2 (*n* = 32)	<0.0001**
Intracerebral hemorrhage	15.7 (*n* = 11)	7.9 (*n* = 35)	<0.04**
Anoxic brain injury	11.4 (*n* = 8)	7.2 (*n* = 32)	0.2
Other	17.1 (*n* = 12)	36.1 (*n* = 160)	<0.005**

*Other diagnoses on admission included severe left ventricular dysfunction, severe tricuspid regurgitation, mitral regurgitation, brain space-occupying lesion, brain abscess, meningitis, acute pancreatitis, amyotrophic lateral sclerosis, other demyelinating diseases of the CNS, and pulmonary embolism.

**P value < 0.05 was defined as statistically significant.

**Table 3:** Outcome endpoints (mean ± SD, %).

	Group 1 (Outreach ICU) (*n* = 70)	Group 2 (open) (*n* = 443)	*P* value
Length of resources utilization minutes (mean ± SD)	20 ± 8.5	77.5 ± 14.7	**<**0.0001*
Weaning success (%)**	38.5 (*n* = 27)	40.6 (*n* = 180)	0.6
Intraoperative complication rate (%)***	2.8 (*n* = 2)	2.03 (*n* = 9)	0.9
Mortality rate (%)^#^	28.5 (*n* = 20)	28.2 (*n* = 125)	0.9

*P value < 0.05 defined as statistically significant. Decreased length of procedure may result in total saving of more operating room time annually.

**Percent of patients successfully weaned from mechanical ventilation on the day of discharge from the hospital.

***Intraoperative complications included two cases of false passage cannulation (group 1) and six cases of intraoperative bleeding, one pneumothorax, and one case of accidental high level of tracheostomy placement (group 2).

^#^In-hospital mortality.

**Table 4:** Economic rationale of PDT technique versus open surgical method in operating room (mean ± SD).

	Group 1 (Outreach ICU) (*n* = 70)	Group 2 (open) (*n* = 443)	*P* value
Staff and operating room resource cost* (US $ per procedure)	70 ± 10	340 ± 20	**<**0.0001*
Tracheostomy set*** cost (US $ per procedure)	201 ± 10	121 ± 10	<0.05*
Fee charges^#^ (US $ per procedure)	67 ± 10	100 ± 10	>0.05
Total balance (US $ per procedure)	338 ± 10	561 ± 10	<0.01*

*Staff and operating room resources have been estimated by cost of staff time per procedure in the operating room and per procedure in the ward. In spite of the significant difference in length of procedure between both study groups (“length of resources utilization”, group 1: 20 ± 8.5 minutes and group 2: 77.5 ± 14.7 minutes) the estimating cost of staff time per procedure was also different.

**P < 0.05 defined as statistically significant.

***Tracheostomy set has relative similarity and homogeneity for every case of open surgical tracheostomy or percutaneous bedside tracheostomy.

^#^The fee charges represent daily municipally resources as water, electricity, and so forth per procedure. This parameter also depends on the time of procedure.
